# Pulmonary sclerosing hemangioma with lymph node metastasis: A case report and literature review

**DOI:** 10.3892/ol.2014.1831

**Published:** 2014-01-28

**Authors:** YASUSHI ADACHI, KOJI TSUTA, RYUJI HIRANO, JIN TANAKA, KEIZO MINAMINO, TOMOHIKO SHIMO, SUSUMU IKEHARA

**Affiliations:** 1 Division of Surgical Pathology, Toyooka Hospital, Toyooka, Hyogo 668-8501, Japan; 2 Department of Stem Cell Disorders, Kansai Medical University, Hirakata, Osaka 573-1010, Japan; 3 Department of Pathology and Clinical Laboratories, National Cancer Center Hospital, Tokyo 104-0045, Japan; 4 Division of Thoracic and Cardiovascular Surgery, Toyooka Hospital, Toyooka, Hyogo 668-8501, Japan; 5 Department of Ophthalmology, Kansai Medical University, Hirakata, Osaka, Japan; 6 Department of Pediatrics, Kansai Medical University, Hirakata, Osaka, Japan

**Keywords:** sclerosing hemangioma, lymph node metastasis, lung, middle-aged female

## Abstract

Pulmonary sclerosing hemangioma (SH) is an uncommon benign or low-grade malignant tumor. Multicentric SH and SH with lymph node metastasis have rarely been reported. The present report describes a case of pulmonary SH with lymph node metastasis in a middle-aged female. A nodule was found incidentally in the lower left lung. The patient underwent left lower pulmonary lobectomy and lymph node dissection. Histologically, the nodule demonstrated the characteristic features of SH and one of the resected lymph nodes contained a metastasis of this tumor. Thus, pulmonary SH has the potential to metastasize, a potential not suggested by histological features.

## Introduction

Sclerosing hemangioma (SH) of the lung is an uncommon tumor that was first described by Leibow and Hubbell in 1956 (1). SH is a lung tumor with a distinctive constellation of histological findings, including solid, papillary, sclerotic and hemorrhagic patterns (2). SH usually presents as a slow-growing benign mass in the lower lobes of middle-aged females (3). Several reports have described multicentric SHs or SHs with lymph node metastasis (4–19). Thus, SH is not always benign and it has the potential to metastasize.

## Case report

### Clinical summary

A 40-year-old female was referred to Toyooka Hospital (Toyooka, Hyogo, Japan) after chest X-ray screening revealed a nodule in the left lower pulmonary field. The patient had no history of smoking. Family history was negative for relevant diseases. Blood tests revealed no increase in concentrations of tumor markers.

**Figure 1:**
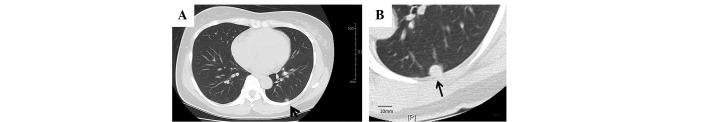
Computed tomography scan images of. (A) whole chest and (B) enlarged scan of the tumor.

Chest computed tomography (CT) scanning revealed a nodule ~10 mm in diameter in the left lower lung (Fig. 1), but no mediastinal or hilar lymph node swelling. The patient underwent lobectomy of the left lower lung with lymph node dissection.

### Pathological findings

**Figure 2:**
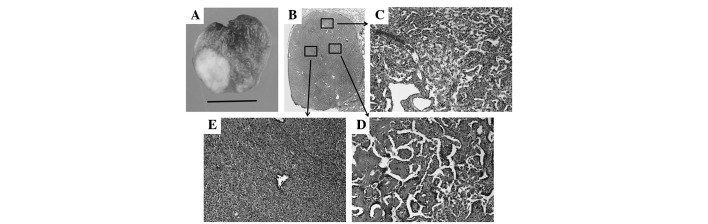
Macroscopic and microscopic findings. (A) Photograph of the tumor; (B) photograph of the tumor stained with hematoxylin and eosin captured through a magnifying glass; and (C–E) various microscopic features of the tumor (magnification, ×20).

Macroscopically, the tumor was sharply demarcated from the surrounding lung tissue and ~10×10×10 mm in size (Fig. 2A). The cut surface was whitish and sclerotic.

Microscopically, the tumor demonstrated various features characteristic of SH, including angiomatoid areas, sclerosis, papillary structures lined with cuboidal cells and sheets of round to polygonal cells with slightly eosinophilic cytoplasms.

**Figure 3:**
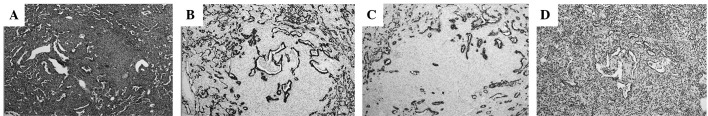
Immunohistological staining with (A) hematoxylin and eosin, (B) anti-napsin A antibody, (C) anti-cytokeratin AE1/AE3 antibody and (D) anti-thyroid transcription factor 1 antibody (magnification, ×20).

Immunohistochemically, the surface-lining cells were positive for napsin A, cytokeratin AE1/AE3 (Fig. 3) and cytokeratin 7 (data not shown). The other cells were negative for these markers. However, all the tumor cells (both the surface-lining and polygonal cells) were positive for thyroid transcription factor 1 (TTF-1), which is expressed not only in thyroid epithelial cells but also in type II pneumocytes and Clara cells, and epithelial membrane antigen. These findings suggested that the tumor was an SH.

**Figure 4:**
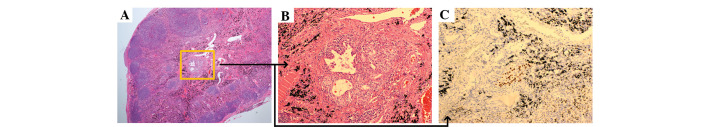
Lymph node metastasis of sclerosing hemangioma. (A) Photograph showing a mediastinal lymph node from the patient. Microscopic evaluation of the metastatic area with (B) hematoxylin and eosin staining and (C) staining with anti-thyroid transcription factor 1 antibody.

A small metastatic focus of SH was identified in one mediastinal lymph node. This lesion shared the papillary pattern of the primary tumor and was TTF-1-positive (Fig. 4). The patient provided written informed consent. This study was approved by the Ethics Committee of Toyooka Hospital (Toyooka, Japan).

After two years of follow-up the patient has not exhibited any recurrence nor metastasis of the tumor.

## Discussion

Pulmonary SH was originally thought to be derived from the endothelium due to histological similarity to cutaneous SH (1).

In the present literature review, PubMed and JDream III (http://jdream3.com/) were used to search for studies written in English or Japanese reporting cases of pulmonary SH with metastasis in the lymph nodes, using the search terms ‘sclerosing hemangioma’, ‘lung’ and ‘metastasis’. The results of these searches returned 17 such cases, of which 13 were in English and 4 in Japanese. Of the 4 studies written in Japanese, 3 cases were abstracts of congresses. Table I lists these cases, including the present report.

Analysis of the data provided in these reports revealed the following about SH with lymph node metastasis: i) The age of the patients ranged between 22 and 67 years [mean ± SD, 36±15 years]; ii) males accounted for 8/17 cases (47.1%) and females 9/17 cases (52.9%); iii) 9/17 (52.9%) primary tumors were found in the left lung and 8/17 (47.1%) were found in the right lung; iv) the left upper lobe was involved in 1/17 cases (5.9%), the left lower lobe in 8 (47.1%), the right upper lobe in 2 (11.8%), the right middle lobe in 1 (5.9%) and the right lower lobe in 5 (29.4%); and v) the primary tumors ranged in size between 10 and 100 mm (mean, 44.8±29.1 mm).

Previously, Devouassoux-Shisheboran *et al* analyzed 100 cases of SH, including one with lymph node metastasis (8). In this study, the clinical and pathological features of these tumors were analyzed in detail. Patients ranged in age between 13 and 76 years (mean, 46 years). There were 83 female and 17 male patients; thus, the female-to-male ratio was 5:1. The left lung was the site of 46% of tumors (17% in the left upper lobe, 25% in the left lower lobe, 1% in the fissure between the upper and lower lobe and the specific site was unknown in 3% of cases), and 54% were found in the right lung (9% in the right upper lobe, 17% in the right middle lobe, 22% in the right lower lobe, 4% in the fissure between the middle and upper lobe, 1% in the fissure between the middle and lower lobe and the specific site was unknown in 1% of cases). The tumors ranged in size between 3 and 70 mm (mean, 26 mm).

In the present study, the cases of SH with lymph node metastases that we compiled were compared with the cases of SH that Devouassoux-Shisheboran *et al* analyzed (8). As shown in Table II, SH with lymph node metastasis tended to occur more often in relatively young male patients than SH without metastasis. The mean size of primary SHs that had lymph node metastasis was larger than the mean size of non-metastatic primary SHs.

The findings that SHs with lymph node metastasis are larger and occur in younger patients may possibly correlate with the more rapid growth of these tumors. However, it is difficult to explain why there is a high frequency of SHs with lymph node metastasis in male patients and in the left lower lobe. Further investigation is required to elucidate the mechanism of metastasis of SH.

## Figures and Tables

**Table I:** Cases of pulmonary sclerosing hemangioma with lymph node metastasis.

			Primary tumor		Metastases			
					
No.	Age, years	Gender	Location	Size, mm	Lymph nodes, n	Maximum size, mm	Location	Reference
1	22	M	R lower	50	1	3	Hilum	6
2	48	M	R lower	80	2	2	Hilum	7
3	ND	ND	ND	35	2	ND	Hilum	8
4	67	F	R lower	90	5	ND	Hilum, mediastinum	9
5	10	F	R middle	47	1	5	Regional	10
6	45	F	R upper	25	3	7	Hilum	10
7	45	M	L lower	37	1	3	Mediastinum	10
8	50	F	L lower	15	1	12	Intralobular	10
9	19	M	L upper (lingula)	30	ND	ND	Intrapulmonary, intralobular	11
10	19	F	L Lower	100	11	ND	Intrapulmonary, interlobular, hilum	12
11	37	F	L lower	20	1	ND	Saltcellar	13
12	35	M	L lower	ND	1	ND	Mediastinum	14
13	23	M	R upper	90	Multiple	ND	Hilum	15
14	24	F	R lower	ND	ND	ND	ND	16
15	35	M	L lower	33	2	ND	Mediastinum	17
16	55	M	R lower	22	1	ND	Intrapulmonary	18
17	38	F	L lower	33	1	ND	Intralobular	19
18	40	F	L lower	10	1	0.5	Mediastinum	PC

F, female; M, male; L, left; R, right; ND, not described; PC, present case.

**Table II:** SH cases and SH cases with lymph node metastasis.

Parameter	SH^a^	SH with lymph node metastasis^b^
Patients, n	100	18
Age, years (mean)	13–76 (46)	22–67 (36±15)
Gender, male : female	1:5	8:9
Primary tumor size, mm (mean)	3–70 (26)	10–100 (44.8±29.1)
Primary tumor location, %		
Left lung	46	53
Right lung	54	47
Left upper lobe	16	6
Left lower lobe	25	48
Right upper lobe	9	11
Right middle lobe	16	6
Right lower lobe	22	29

a SH cases analyzed by Devouassoux-Shisheboran *et al* (2);

b SH cases with lymph node metastasis analyzed in the present study.

SH, sclerosing hemangioma.
